# Syntax-Guided Quantifier Instantiation

**DOI:** 10.1007/978-3-030-72013-1_8

**Published:** 2021-02-26

**Authors:** Aina Niemetz, Mathias Preiner, Andrew Reynolds, Clark Barrett, Cesare Tinelli

**Affiliations:** 8 Eindhoven University of Technology, Eindhoven, The Netherlands; 9 Aalborg University, Aalborg East, Denmark; 10 Stanford University, Stanford, USA; 11 The University of Iowa, Iowa City, USA

## Abstract

This paper presents a novel approach for quantifier instantiation in Satisfiability Modulo Theories (SMT) that leverages syntax-guided synthesis (SyGuS) to choose instantiation terms. It targets quantified constraints over background theories such as (non)linear integer, reals and floating-point arithmetic, bit-vectors, and their combinations. Unlike previous approaches for quantifier instantiation in these domains which rely on theory-specific strategies, the new approach can be applied to any (combined) theory, when provided with a grammar for instantiation terms for all sorts in the theory. We implement syntax-guided instantiation in the SMT solver CVC4, leveraging its support for enumerative SyGuS. Our experiments demonstrate the versatility of the approach, showing that it is competitive with or exceeds the performance of state-of-the-art solvers on a range of background theories.

## 1 Introduction

Modern Satisfiability Modulo Theories (SMT) solvers are highly efficient tools, capable of reasoning about constraints over a wide range of logical theories, including (non-linear) real and integer arithmetic, fixed-size bit-vectors, and floating-point arithmetic. Their core algorithms are designed primarily for quantifier-free constraints, but various extensions have been shown to work well also for quantified constraints in many cases. Quantified reasoning in SMT has many practical applications, including software verification, automated theorem proving, and synthesis.

Current SMT solvers handle quantified constraints in a variety of ways, with a degree of effectiveness that usually depends on the background theory. For instance heuristic instantiation techniques such as E-matching [15] are used for quantified formulas with heavy use of uninterpreted functions. These heuristic instantiation techniques are refutationally incomplete but they can be highly effective, in particular in the context of verification applications. For quantified constraints over a particular background theory, such as linear arithmetic or fixed-size bit-vectors, on the other hand, SMT solvers resort to an entirely different set of techniques. While also based on quantifier instantiation, these other

* This work was supported in part by DARPA (award no. FA8650-18-2-7861), NSF (award no. 1656926) and ONR (award no. N68335-17-C-0558).

techniques tend to be counterexample-guided and can be complete for theories and fragments of first-order logic that admit quantifier elimination.

Specific previous work in the latter direction includes counterexample-guided quantifier instantiation techniques for linear arithmetic [25] and fixed-size bit-vectors [18,20]. The key to developing each of them is to devise an appropriate, theory-specific *selection function*, which determines a term selection strategy for instantiating universal quantifiers. For some logics, e.g., linear arithmetic, selection functions can be based on the notion of elimination set found in classic algorithms for quantifier elimination [9,14]. However, since many theories used in practice do not admit quantifier elimination, the design of a good selection function is usually non-trivial. These challenges are further magnified when reasoning in combinations of multiple theories.

We propose a novel, *syntax-guided quantifier instantiation (SyQI)* approach, which is both general-purpose and highly effective for quantified formulas in background theories such as (non)linear integer, reals and floating-point arithmetic, and their combinations. The new approach leverages an embedding of a solver for the syntax-guided synthesis (SyGuS) problem [1] within an SMT solver in order to choose terms for quantifier instantiation in a counterexample-guided manner. It is theory-agnostic and only requires the specification, via a grammar, of the set of terms to consider for each sort in the theory when instantiating quantifiers.[3] Since it can be applied to quantified formulas in any background theory, it is more general in scope than previous work [20]. Our approach is intended for logics such as quantified floating-point arithmetic, which would benefit from counterexample-guided quantifier instantiation, but for which appropriate selection function are not obvious. We show that the use of syntax-guided synthesis gives us the flexibility to develop variants of our approach that are highly competitive with the state of the art in SMT solving. More specifically, this paper makes the following *contributions*:

- We present and prove correct a simple yet novel quantifier instantiation approach that leverages syntax-guided synthesis for selecting instantiations.
- We explore variants of the approach along several dimensions, including the choice of symbols to include in grammars for various background theories.
- We implement this technique in the SMT solver CVC4 [5] and show that it performs remarkably well in a wide variety of SMT logics. In particular, it improves upon the state of the art for solving quantified formulas over floating-point arithmetic, and is highly competitive for non-linear integer arithmetic and certain combined logics that involve fixed-size bit-vectors.

*Related Work.* Handling quantified formulas in SMT solvers is a long-standing challenge. Early approaches for quantified formulas were largely based on E-matching [8,10,15]. They have been later supplemented with techniques that rely on models for establishing satisfiability [11,26], and on conflict finding to accelerate the search for unsatisfiability [27]. Pragmatic enumerative approaches

[3] Our implementation provides a default grammar for all supported sorts. In general, grammars can also be provided by the user. We do not explore this option here.

for quantifier instantiation have also been explored and shown to increase the precision of SMT solvers on inputs involving uninterpreted functions where E-matching is incomplete [21]. The approach we describe here is also enumerative in nature; however, it leverages syntax-guided synthesis for choosing instantiations and does not target inputs with uninterpreted functions.

For quantified formulas over a single background theory, counterexample-guided approaches have been considered by Bjørner and Janota [6] and by Reynolds et al. [25], targeting primarily quantified linear integer/real arithmetic. For theories of other data types (e.g., bit-vectors), most approaches use *value-based* instantiation, where concrete variable assignments for a set of quantifier-free formulas derived from the negation of the input formula (the counterexamples) provide instantiations for the universal variables. In the SMT solver Z3 [16], model-based quantifier instantiation (MBQI) [11] is combined with a template-based model finding procedure [29]. A recent line of work by Niemetz et al. [18] leverages invertibility conditions in a counterexample-guided loop for quantifier instantiation of formulas in the theory of fixed-size bit-vectors. Brain et al. [7] lift the concept of invertibility conditions to the theory of floating-point arithmetic and presented a preliminary quantifier elimination procedure for a fragment of the theory based on these conditions. Another approach for lazy quantifier elimination for bit-vector formulas is explored by Vediramana Krishnan et al. [12], based on iterative approximate quantifier elimination.

Reynolds et al. [24] leverage counterexample-guided quantifier instantiation (CEGQI) to efficiently solve a restricted but practically useful form of syntax-guided synthesis problems. In contrast, the work in this paper has the dual goal of leveraging enumerative syntax-guided synthesis to establish a strategy for quantifier instantiation of (first-order) quantified formulas.

SyGuS techniques to solve quantified problems were previously explored by Preiner et al. in [20]. However, instead of focusing on quantifier instantiation they combined enumerative syntax-guided synthesis with value-based quantifier instantiation to synthesize Skolem functions for existential variables.

## 2 Background

We assume the usual notions and terminology of many-sorted first-order logic with equality (denoted by $\approx$). Let $S$ be a set of *sort symbols*. For every $\sigma \in S$, let $X_\sigma$ be an infinite set of *variables of sort* $\sigma$. Let $X = \bigcup_{\sigma \in S} X_\sigma$. Let $\Sigma$ be a *signature* consisting of a set $\Sigma^s \subseteq S$ of sort symbols and a set $\Sigma^f$ of interpreted (and sorted) function symbols $f^{\sigma_1 \cdots \sigma_n \sigma}$ with arity $n \geq 0$ and $\sigma_1, ..., \sigma_n, \sigma \in \Sigma^s$. We assume that $\Sigma$ includes a Boolean sort $\mathsf{Bool}$ and the Boolean values $\top$ (true) and $\bot$ (false). Let $\mathcal{I}$ be a *$\Sigma$-interpretation* that maps: each sort $\sigma \in \Sigma^s$ to a non-empty set $\sigma^{\mathcal{I}}$ (the *domain* of $\mathcal{I}$), with $\mathsf{Bool}^{\mathcal{I}} = \{\top, \bot\}$; each variable $x \in X_\sigma$ to an element $x^{\mathcal{I}} \in \sigma^{\mathcal{I}}$; and each function $f^{\sigma_1 \cdots \sigma_n \sigma} \in \Sigma^f$ to a total function $f^{\mathcal{I}}\colon \sigma_1^{\mathcal{I}} \times ... \times \sigma_n^{\mathcal{I}} \rightarrow \sigma^{\mathcal{I}}$ if $n > 0$, and to an element in $\sigma^{\mathcal{I}}$ if $n = 0$.

We assume the usual definition of well-sorted terms, literals, and formulas as $\mathsf{Bool}$ terms with variables in $X$ and symbols in $\Sigma$, and refer to them as $\Sigma$-

terms, $\Sigma$-atoms, and so on. A *ground* term/formula is a $\Sigma$-term/formula without variables. We define $\boldsymbol{x} = (x_1, ..., x_n)$ as a tuple of variables and write $Q\boldsymbol{x}.\varphi$ with $Q \in \{\forall, \exists\}$ for a *quantified* formula $Qx_1.\cdots Qx_n.\varphi$. A formula is *universal* if it has the form $\forall \boldsymbol{x}. P$ where $P$ is a quantifier-free formula. For simplicity, *we consider only universal quantifiers* since existential quantifiers can be rewritten in terms of universal ones. We use $\text{Lit}(\varphi)$ to denote the set of $\Sigma$-literals of $\Sigma$-formula $\varphi$. For a $\Sigma$-term or $\Sigma$-formula $e$, we use $e[\boldsymbol{x}]$ to indicate that the free variables of $e$ are in $\boldsymbol{x}$. For a tuple of $\Sigma$-terms $\boldsymbol{t} = (t_1, ..., t_n)$, we write $e[\boldsymbol{t}]$ for the term or formula obtained from $e$ by simultaneously replacing each occurrence of $x_i$ in $e$ by $t_i$. If $t$ is a $\Sigma$-term/formula and $\mathcal{I}$ a $\Sigma$-interpretation, we write $t^{\mathcal{I}}$ to denote the meaning of $t$ in $\mathcal{I}$. We use the usual inductive definition of a satisfiability relation $\models$ between $\Sigma$-interpretations and $\Sigma$-formulas.

A *theory* $T$ is a pair $(\Sigma, I)$, where $\Sigma$ is a signature and $I$ is a non-empty class of $\Sigma$-interpretations (the *models* of $T$) that is closed under variable reassignment, i.e., every $\Sigma$-interpretation that only differs from an $\mathcal{I} \in I$ in how it interprets variables is also in $I$. A $\Sigma$-formula $\varphi$ is $T$*-satisfiable* (resp. $T$*-unsatisfiable*) if it is satisfied by some (resp. no) interpretation in $I$; it is $T$*-valid* if it is satisfied by all interpretations in $I$.

*Enumerative SyGuS using an Embedding into Datatypes.* A syntax-guided synthesis problem for an $n$-ary function $f$ in a background theory $T$ consists of a set of semantic restrictions (a *specification*) for $f$, given as a (second-order) $T$-formula of the form $\exists f.\, \varphi[f]$, and a set of syntactic restrictions on the solutions for $f$, typically expressed as a *context-free grammar*. A solution to such a problem is a term $t[x_1, \ldots, x_n]$ that satisfies the syntactic restrictions and is such that the formula $\varphi[\lambda x_1, \ldots, x_n.t]$ is $T$-valid.

As shown in previous work [24], syntactic restrictions for the bodies of functions to synthesize can be conveniently represented as a set of *(algebraic) datatypes*. The setting in this paper is simpler. Instead of synthesizing terms corresponding to function bodies, we use context-free-grammars for defining a set of (first-order) terms in a given theory, possibly containing free function symbols. For instance, let $a$ and $b$ be free constants of sort $\mathsf{Int}$. The context-free grammar $R$ below specifies a set of integer ($Z$) and Boolean ($B$) terms:

$$Z ::= 0 \mid 1 \mid a \mid b \mid Z + Z \mid Z - Z \mid \mathsf{ite}(B, Z, Z) \quad (1)$$

$$B ::= B \geq B \mid Z \approx Z \mid \neg B \mid B \wedge B \quad (2)$$

Given such a grammar, our SyGuS solver generates the following mutually recursive datatypes:

$$\mathcal{Z} = \mathsf{zero} \mid \mathsf{one} \mid \mathsf{a} \mid \mathsf{b} \mid \mathsf{plus}(\mathcal{Z}, \mathcal{Z}) \mid \mathsf{minus}(\mathcal{Z}, \mathcal{Z}) \mid \mathsf{ite}(\mathcal{B}, \mathcal{Z}, \mathcal{Z}) \quad (3)$$

$$\mathcal{B} = \mathsf{geq}(\mathcal{Z}, \mathcal{Z}) \mid \mathsf{eq}(\mathcal{Z}, \mathcal{Z}) \mid \mathsf{not}(\mathcal{B}) \mid \mathsf{and}(\mathcal{B}, \mathcal{B}) \quad (4)$$

Each datatype constructor, listed on the right-hand side of each equation, corresponds to a production rule of $R$, e.g., $\mathsf{plus}$ corresponds to the rule $Z ::= Z + Z$. Given a datatype value $v$, we write $\textbf{to\_term}(v)$ to denote the term that $v$ represents, e.g., $\textbf{to\_term}(\mathsf{plus}(\mathsf{a}, \mathsf{b}))$ is the term $a + b$.

In previous work [22, 24], a *smart* enumerative approach for syntax-guided synthesis was presented and implemented in CVC4. In that work, the generation of terms is based on finding solutions for an evolving set of constraints in an extension of the quantifier-free fragment of algebraic datatypes, for which some SMT solvers have dedicated decision procedures [3, 23]. In the remainder of this paper, we write $T_{\mathsf{D}}$ to denote the theory of datatypes over a signature $\Sigma_{\mathsf{D}}$ of *constructor* and *selector* symbols. The signature $\Sigma_{\mathsf{D}}$ includes (parametric) datatype sorts that are interpreted as the universe of a term algebra over the constructors. Selectors are interpreted as functions that extract the immediate subterms of a constructor term.

In our setting, datatype constraints are used to express syntactic restrictions on the terms in the original theory. For instance, in case of the example theory and corresponding datatypes $\mathcal{Z}$ and $\mathcal{B}$ defined above, we can write a datatype constraint that is falsified by all terms of the form $\mathsf{plus}(\mathsf{zero}, t)$ where $t$ is a constructor term of sort $\mathcal{Z}$. This corresponds to ruling out terms of the form $(0 + \ldots)$ in the original theory where $s$ is a term of sort $\mathsf{Int}$. In more detail, for a datatype term $d$, we write $\mathsf{is}_{\mathsf{C}}(d)$ to denote the *discriminator* predicate, which is satisfied exactly when $d$ is interpreted as a datatype value whose top constructor is $\mathsf{C}$. We write $\mathsf{sel}_{\sigma,n}(d)$ to denote a *shared selector* [28] applied to $d$, interpreted as the $n^{th}$ child of $d$ with sort $\sigma$ if one exists, and as an arbitrary element of $\sigma$ otherwise. These symbols are used for constructing *blocking constraints.* For example, we can write $\neg\mathsf{is}_{\mathsf{plus}}(d) \vee \neg\mathsf{is}_{\mathsf{zero}}(\mathsf{sel}_{\mathcal{Z},1}(d))$ to state the constraint above that $d$ cannot be interpreted as any datatype value corresponding to an $\mathsf{Int}$ term of the form $(0 + \ldots)$. In the context of syntax-guided synthesis, a constraint like this is added, for instance, to filter out redundant terms (like $0 + \ldots$) or terms already known to falsify the synthesis conjecture.

Our approach for syntax-guided instantiation relies on a notion of *evaluation variables.* A related, more general, notion of evaluation functions was used in the context of syntax-guided synthesis (see Section 2 of [22] for details). Let $d$ be a term of a datatype sort encoding a grammar over terms of sort $\sigma$. We write $\mathsf{e}_d$ to denote a free constant of sort $\sigma$, which we call the evaluation variable for $d$. We use evaluation variables to determine which terms to use in instantiations of quantified formulas. The algorithm given in the following section will add constraints that force the interpretation of $\mathsf{e}_d$ to be equal to $\mathbf{to\_term}(d^{\mathcal{I}})$ in interpretations $\mathcal{I}$. A simple example of such a constraint is $\mathsf{is}_{\mathsf{a}}(d) \Rightarrow \mathsf{e}_d \approx a$, stating that the evaluation variable $\mathsf{e}_d$ for $d$ is equal to the free constant $a$ of integer type when $d$ is interpreted as the datatype value $\mathsf{a}$.

## 3 SyGuS Quantifier Instantiation (SyQI)

Our new SyGuS-based instantiation approach combines counterexample-guided quantifier instantiation (CEGQI) with smart enumerative SyGuS techniques to synthesize terms for quantifier instantiation. In essence, it is an algorithm that tries to synthesize a term $t$ for a variable $x$ in a given formula $\forall x.\, P[x]$ such that $\neg P[t]$ holds. For synthesis purposes, each quantified variable is associated with

**Algorithm 1** Main algorithms of the SyQI approach.

1: **procedure syqi**($\{Q_1, \ldots, Q_n\}, G$)
2:     **for** $Q_j \in \{Q_1, \ldots, Q_n\}$ with $Q_j = \forall \boldsymbol{x}.\, P[\boldsymbol{x}]$ **do**
3:         **for** $x \in \boldsymbol{x}$ **do**
4:             Let $d_x$ be a fresh global constant of datatype sort $\mathbf{grammar}_{\mathcal{S}}(x)$
5:         $G \coloneqq G \cup \{l_j \Rightarrow \neg P[\mathsf{e}_{\boldsymbol{d_x}}]\}$ with fresh Boolean constant $l_j$ and fresh $\mathsf{e}_{d_x}$
6:     **repeat**
7:         **if check**($G$) $=$ *unsat* **then return** *unsat*
8:         $r, \mathcal{I} \coloneqq$ **check**($G \wedge (l_1 \vee \ldots \vee l_n)$)
9:         **if** $r =$ *unsat* **then return** *sat*
10:         **for** $l_j \in \{l_1, \ldots, l_n\}$ such that $l_j^{\mathcal{I}} = \top$ **do**
11:             $G \coloneqq G \cup$ **select_lemmas**$_{\mathcal{L}}(Q_j, \mathcal{I})$

12: **procedure select_lemmas**$_{\mathcal{L}}$($\forall x_1, \ldots, x_p.\, P[x_1, \ldots, x_p], \mathcal{I}$)
13:     $L \coloneqq \emptyset$
14:     **for** $x_i \in \{x_1, \ldots, x_p\}$ **do**
15:         $t_i \coloneqq$ **to_term**($d_{x_i}^{\mathcal{I}}$)
16:         $L \coloneqq L \cup \{$**explain**$(d_{x_i} \approx d_{x_i}^{\mathcal{I}}) \Rightarrow \mathsf{e}_{d_{x_i}} \approx$ **to_term**$(d_{x_i}^{\mathcal{I}})\}$
17:     **return** non-empty subset of $\{P[t_1, \ldots, t_p]\} \cup L$ based on selection strategy $\mathcal{L}$

a SyGuS grammar based on the sort of the variable. For example, our algorithm uses a bit-vector-specific grammar to synthesize bit-vector terms as possible instantiations of quantified variables of bit-vector sort. Our SyGuS solver suggests instantiations based on such grammars and an evolving set of constraints on the instance term. The main advantage of this instantiation approach is that it does not require theory-specific quantifier instantiation algorithms. Its only theory-specific aspects are the construction of the grammar for each theory sort and the satisfiability checks performed on the generated instances.

Algorithm 1 shows the two main procedures **syqi** and **select_lemmas**$_{\mathcal{L}}$ of our SyGuS instantiation approach. To simplify the exposition, we describe the restricted case where the quantified input formula are all universal. Our implementation in CVC4, however, applies to the general case through a lazy conversion to DNF and resolution of quantifier alternations.

Procedure **syqi** takes as argument a set $\{Q_1, \ldots, Q_n\}$ of *universal* (quantified) $T$-formulas and a set $G$ of *ground* $T$-formulas. As an initial step, and prior to solving the problem, we generate a lemma for each quantified formula $Q_i$ as part of our counterexample-guided quantifier instantiation approach (lines 2-5). We first create a fresh datatype constant $d_x$ of sort $\mathbf{grammar}_{\mathcal{S}}(x)$ for each variable $x \in \boldsymbol{x}$ in each input formula $\forall \boldsymbol{x}.\, P[\boldsymbol{x}]$. The datatype sort $\mathbf{grammar}_{\mathcal{S}}(x)$ is constructed from a SyGuS grammar determined by the sort of variable $x$. The language generated by the grammar includes ground terms from $Q_i$ and $G$ of the same sort. These terms are chosen following a selection strategy $\mathcal{S}$, which we describe in Section 3.1. Apart from running **check**, used as a black box, $\mathbf{grammar}_{\mathcal{S}}$ implements the only theory-specific handling of our procedure. Finally, we add to $G$ a lemma of the form $l_i \Rightarrow \neg P[\mathsf{e}_{\boldsymbol{d_x}}]$ for each quantified formula, where $l_i$ is a fresh Boolean constant (the counterexample literal for $Q_i$). Thanks to $l_i$ being fresh, this preserves the satisfiability of $G$. The notation $\mathbf{e}_{\boldsymbol{d_x}}$ is a shorthand for $(\mathsf{e}_{d_{x_1}}, \ldots, \mathsf{e}_{d_{x_m}})$, the tuple of evaluation variables for each $d_x$ of $x \in \boldsymbol{x}$. The purpose of a counterexample lemma is twofold. First, it indicates whether a quantified formula $Q_i$ is *active* ($l_i$ assigned to true) or *inactive* ($l_i$ assigned to false). Second, it focuses on finding counterexamples that falsify the body of $Q_i$.

The main loop of procedure **syqi** is provided in lines 6-11. Each iteration starts with a quantifier-free satisfiability check (performed by procedure **check** on line 7) on the current set of ground formulas $G$ in the combined theory $T \cup T_{\mathsf{D}}$. If $G$ is unsatisfiable, procedure **syqi** returns *unsat*. If $G$ is satisfiable, the procedure further checks whether it can find a counterexample for any of the quantified formulas $Q_1, \ldots, Q_n$, which is done by checking the satisfiability of $G \wedge (l_1 \vee \ldots \vee l_n)$. If the check returns *unsat* then no more counterexamples can be found; the algorithm concludes that input set is satisfiable and returns *sat*. The reason is that, in this case, the set $G$ is satisfiable and entails each input formula, as proven later in this section. If the second call to **check** (line 8) returns *sat*, it additionally returns (a finite representation of) a model $\mathcal{I}$ for the current set of ground formulas $G$. Since $\mathcal{I}$ satisfies $l_1 \vee \ldots \vee l_n$, it does not satisfy at least one quantified formula in $Q_1, \ldots, Q_n$.[4] For each active quantified formula in $\mathcal{I}$, we generate new lemmas via procedure **select_lemmas**$_{\mathcal{L}}$ (lines 10-11), and repeat the main loop of the algorithm. Note that the second satisfiability check can be avoided by employing a special decision heuristic for counterexample literals $l_i$ in the SAT solver. The decision heuristic will always assign a counterexample literal $l_i$ to true on a decision. Consequently, $l_i$ can only be assigned to false in a candidate interpretation $\mathcal{I}$ if $\neg l_i$ is entailed by the set of ground formulas $G$.

Procedure **select_lemmas**$_{\mathcal{L}}$ takes a formula $\forall \boldsymbol{x}.\, P[\boldsymbol{x}]$ and a model $\mathcal{I}$ as arguments and generates a set of lemmas based on $\mathcal{I}$ and selection strategy $\mathcal{L}$. The procedure maintains the invariant of always returning a set of lemmas $L$ where $L \setminus G$ is non-empty. This set $L$ includes a single *instantiation lemma* (of the form $P[\boldsymbol{t}]$) and an *evaluation unfolding lemmas* (see below) for each variable $x \in \boldsymbol{x}$. The returned lemmas are generated based on one of three *lemma selection strategies*: priority-inst, priority-eval, and interleave. Strategy interleave selects both the instantiation lemma and a set of evaluation unfolding lemmas at the same time. Strategies priority-inst and priority-eval give priority to instantiation lemmas and evaluation unfolding lemmas, respectively; i.e., strategy priority-inst selects the instantiation lemma and only selects evaluation unfolding lemmas if the instantiation lemma was already in $G$. Analogously, priority-eval gives priority to evaluation unfolding lemmas.

The various lemmas are constructed as follows. For each variable $x \in \boldsymbol{x}$ we use the model value $d_x^{\mathcal{I}}$ of datatype constant $d_x$ to construct the corresponding term **to_term**$(d_x^{\mathcal{I}})$ in the theory of variable $x$ (line 15). The constructed term corresponds to a term synthesized by the SyGuS extension of our datatypes

[4] Note that this does not mean the quantified formula is unsatisfiable, only that it is not satisfied in $\mathcal{I}$.

solver based on the grammar specified for $x$. To ensure that $d_x$ evaluates to the same values as term $\textbf{to\_term}(d_x^{\mathcal{I}})$ under model value $d_x^{\mathcal{I}}$, we generate the evaluation unfolding lemma $\textbf{explain}(d_x \approx d_x^{\mathcal{I}}) \Rightarrow \mathsf{e}_{d_x} \approx \textbf{to\_term}(d_x^{\mathcal{I}})$. The explanation for the model value $d_x^{\mathcal{I}}$ is expressed in terms of discriminator predicates. For example, if value $d_x^{\mathcal{I}}$ represents term $a + b$, the procedure generates lemma $\mathsf{is}_{\mathsf{plus}}(d_x) \wedge \mathsf{is}_{\mathsf{a}}(\mathsf{sel}_{\mathcal{Z},1}(d_x)) \wedge \mathsf{is}_{\mathsf{b}}(\mathsf{sel}_{\mathcal{Z},2}(d_x)) \Rightarrow \mathsf{e}_{d_x} = a + b$. As a last step, $\textbf{select\_lemmas}_{\mathcal{L}}$ selects a non-empty subset of the generated instantiation lemma $P[t_1, \ldots, t_p]$ (where each $t_i$ is $\textbf{to\_term}(d_{x_i}^{\mathcal{I}})$) and the evaluation unfolding lemmas $L$ according to the lemma selection strategy $\mathcal{L}$.

We now discuss the correctness properties of our approach. In the following, we say a grammar $R$ for sort $\sigma$ is *complete*, if for all interpretations $\mathcal{I}$ and values $v$ of sort $\sigma$, it generates at least one term $t$ such that $t^{\mathcal{I}} = v$. Note that we only consider complete grammars in this paper. We say a lemma selection strategy $\mathcal{L}$ is *fair* wrt a set of formulas $G$ if it returns a set of lemmas that contain at least one lemma inequivalent to each formula in $G$ whenever such lemma exists.

**Theorem 1.** *Let $T$ be a theory with signature $\Sigma$, let $F$ be a set of universal formulas $\{Q_1, \ldots, Q_n\}$ and $G_0$ is a set of quantifier-free formulas. If all grammars constructed by the calls to* $\textbf{\textit{grammar}}_{\mathcal{S}}$ *in* ***syqi*** *are complete and the selection strategy $\mathcal{L}$ used for* $\textbf{\textit{select\_lemmas}}_{\mathcal{L}}$ *is fair, then the following statements hold:*

1. *(Refutation Soundness) If* ***syqi***$(F, G_0)$ *returns unsat, $F \cup G_0$ is $T$-unsatisfiable.*
2. *(Model soundness) If* ***syqi***$(F, G_0)$ *returns sat, $F \cup G_0$ is $T$-satisfiable.*
3. *(Progress) Let $G_i$ be the current state of the set of ground formulas $G$ after $i$ iterations of* ***syqi*** *(lines 6-11). Each iteration $i + 1$ adds at least one new formula to $G_i$, so that $G_{i+1} \setminus G_i \neq \emptyset$.*

Conceptually, the proof of refutational soundness relies on the fact that all lemmas added to $G$ are entailed by the input or maintain equisatisfiability with respect to the input. The proof of model soundness relies on the fact that when $G$ collectively entails the negation of (all) quantified formulas, then the current model $\mathcal{I}$ for $G$ must be a model for all quantified formulas. Procedure **syqi** is not terminating in general. However, the progress property guarantees that the algorithm does not get stuck in a single state and keeps making progress towards refining the set of possible models by ruling out at least one candidate model at each iteration of the procedure's main loop.

*Proof.* For brevity, we show these statements for the case of $n = 1$ and where $Q_1$ is $\forall \boldsymbol{x}.\, P[\boldsymbol{x}]$; the proof can be easily lifted to $n > 1$. When **syqi**$(F, G_0)$ terminates, the internal set $G$ is the union of:

- The initial quantifier-free formula $G_0$,
- The counterexample lemma $G_{cex}$ of the form $l \Rightarrow \neg P[\mathbf{e}_{\boldsymbol{d_x}}]$ added on line 5,
- A set of instantiations $G_{inst}$ of the form $P[\boldsymbol{t}]$, and
- A set of evaluation lemmas $G_{ev}$ of the form $C[d] \Rightarrow \mathsf{e}_d \approx t$.

To show (1), assume that $\varphi$ is satisfied by some $\Sigma$-interpretation $\mathcal{J}$, where without loss of generality assume that $l^{\mathcal{J}}$ is false. Let $\mathcal{I}$ be a $\Sigma \cup \Sigma_{\mathsf{D}}$-interpretation that extends $\mathcal{J}$ such that for each evaluation variable $\mathsf{e}_d$, the interpretation of $d$ in $\mathcal{I}$ is such that $\mathbf{to\_term}(d^{\mathcal{I}})^{\mathcal{I}} = \mathsf{e}_d^{\mathcal{I}}$. Such a value exists since our grammars are complete by assumption. We show that $\mathcal{I}$ satisfies each formula $\psi$ in $G$. If $\psi \in G_0$, then this holds since $\mathcal{J}$ satisfies $\varphi$, and hence, by extension $\mathcal{I}$ does as well. If $\psi \in G_{cex}$, then $\psi$ is satisfied by $\mathcal{I}$ since it interprets $l_i$ as false. If $\psi \in G_{inst}$ is an instantiation lemma of some $Q_i$, then it is satisfied by $\mathcal{I}$ since $\mathcal{J}$ also satisfies $Q_i$. If $\psi \in G_{ev}$ is an evaluation lemma, this is satisfied by our construction of $d^{\mathcal{I}}$. Thus $\varphi$ is $T$-satisfiable, then $G$ must be $(T \cup T_{\mathsf{D}})$-satisfiable. Thus, since $\mathbf{syqi}(F, G_0)$ returns *unsat* when $G$ is $(T \cup T_{\mathsf{D}})$-unsatisfiable, this means that $F \cup G_0$ must be $T$-unsatisfiable as well.

To show (2), if $\mathbf{syqi}(F, G_0)$ returns *sat*, then the set $G$ is satisfied by some $\Sigma \cup \Sigma_{\mathsf{D}}$-interpretation and $G \cup \{l_1\}$ is unsatisfiable. Let $\mathcal{J}$ be the $\Sigma$-interpretation that interprets all symbols in $\Sigma$ the same as in $\mathcal{I}$. Since $G \cup \{l_1\}$ is unsatisfiable, we have that $G_0 \cup G_{inst} \cup G_{ev} \cup \{\neg P[\mathbf{e}_{\boldsymbol{d_x}}]\}$ is $T \cup T_{\mathsf{D}}$-unsatisfiable. Since all $\Sigma$-interpretations can be lifted to a $\Sigma \cup \Sigma_{\mathsf{D}}$-interpretation satisfying $G_{ev}$, it must also be the case that $G_0 \cup G_{inst} \cup \{\neg P[\mathbf{e}_{\boldsymbol{d_x}}]\}$ is $T$-unsatisfiable. Hence, all models of $G_0 \cup G_{inst}$ must make $P[\mathbf{e}_{\boldsymbol{d_x}}]$ true. Since $\mathbf{e}_{\boldsymbol{d_x}}$ does not occur in $G_0 \cup G_{inst}$, this implies that all models of $G_0 \cup G_{inst}$ satisfy $\forall \boldsymbol{x}.\, P[\boldsymbol{x}]$. Since $G_0 \cup G_{inst} \subseteq G$ and $\mathcal{I}$ satisfies $G$, we have that $\mathcal{J}$ satisfies $\{\forall \boldsymbol{x}. P[\boldsymbol{x}]\} \cup G$.

To show (3), assume *ad absurdum* that $G$ is satisfied by a $T \cup T_{\mathsf{D}}$-interpretation $\mathcal{I}$ where $\mathbf{to\_term}(\boldsymbol{d_x}^{\mathcal{I}}) = \boldsymbol{t}$ and $Q_1$ is active in $\mathcal{I}$. Also assume that $G$ contains the evaluation unfolding lemmas for $\boldsymbol{d_x}^{\mathcal{I}}$ and the instantiation lemma $P[\boldsymbol{t}]$. Due to the former, we have that $\mathbf{e}_{\boldsymbol{d_x}}{}^{\mathcal{I}} = \boldsymbol{t}^{\mathcal{I}}$. Since $Q_1$ is active in $\mathcal{I}$, $\mathcal{I}$ satisfies $\neg P[\mathbf{e}_{\boldsymbol{d_x}}]$. However, $P[\boldsymbol{t}]$ is also satisfied by $\mathcal{I}$, a contradiction. Thus, at least one of the lemmas returned by $\mathbf{select\_lemmas}_{\mathcal{L}}$ for $Q_1$ must be inequivalent to the lemmas in $G$, due to our assumption that $\mathcal{L}$ is a fair selection strategy. □

### 3.1 Grammar Construction

For quantifier instantiation, we focus on the theories of fixed-size bit-vectors, floating-point numbers, integers, and reals as defined by the SMT-LIB 2 standard [4]. The signature of the theory of fixed-size bit-vectors includes a unique sort for each positive bit-vector width $n$, denoted here as $\mathsf{BV}_{[n]}$. The signature of the theory of floating-point numbers includes a rounding-mode sort $\mathsf{RM}$ and a unique floating-point sort for each combination of positive exponent width $e$ and significand width $s$, denoted here as $\mathsf{FP}_{[e,s]}$. The theories of Integers and Reals include the integer sort $\mathsf{Int}$ and the real sort $\mathsf{Real}$, respectively. For each of these sorts we define a SyGuS grammar that includes the following operators and constants.

$$R_{\mathrm{BV}} : \{\sim, -, \&, |, \oplus, +, \cdot, \div, \div_s, \mathrm{mod}, \mathrm{mod}_s \ll, \gg, \gg_a, 0, 1, \mathrm{ones}, \mathrm{smin}, \mathrm{smax}\}$$

$$R_{\mathrm{FP}} : \{-, \mathrm{abs}, \mathrm{rem}, \surd, \mathrm{rti}, +, \cdot, \div, \mathrm{fma}, \mathrm{NaN}, \pm\infty, \pm 0, \pm\mathrm{min}^s, \pm\mathrm{max}^s, \pm\mathrm{min}^n, \pm\mathrm{max}^n\}$$

$$R_{\mathrm{RM}} : \{\mathrm{RNA}, \mathrm{RNE}, \mathrm{RTE}, \mathrm{RTP}, \mathrm{RTZ}\} \qquad R_{\mathrm{Int}} : \{+, -, 0, 1\} \qquad R_{\mathrm{Real}} : \{+, -, \div, 0, 1\}$$

| Theory | Symbol | SMT-LIB Syntax | Sort |
|---|---|---|---|
| BV | $\sim$, $-$ | bvnot, bvneg | $\mathsf{BV}_{[n]} \to \mathsf{BV}_{[n]}$ |
| | &, \|, $\oplus$ | bvand, bvor, bvxor | $\mathsf{BV}_{[n]} \times \mathsf{BV}_{[n]} \to \mathsf{BV}_{[n]}$ |
| | $\ll$, $\gg$, $\gg_a$ | bvshl, bvlshr, bvashr | $\mathsf{BV}_{[n]} \times \mathsf{BV}_{[n]} \to \mathsf{BV}_{[n]}$ |
| | $+$, $-$, $\cdot$ | bvadd, bvsub, bvmul | $\mathsf{BV}_{[n]} \times \mathsf{BV}_{[n]} \to \mathsf{BV}_{[n]}$ |
| | $\div$, $\div_s$, mod, $\text{mod}_s$ | bvudiv, bvsdiv, bvurem, bvsrem | $\mathsf{BV}_{[n]} \times \mathsf{BV}_{[n]} \to \mathsf{BV}_{[n]}$ |
| FP | $-$,abs | fp.neg, fp.abs | $\mathsf{FP}_{[e,s]} \to \mathsf{FP}_{[e,s]}$ |
| | rem | fp.rem | $\mathsf{FP}_{[e,s]} \times \mathsf{FP}_{[e,s]} \to \mathsf{FP}_{[e,s]}$ |
| | $\sqrt{}$, rti | fp.sqrt, fp.roundToIntegral | $\mathsf{RM} \times \mathsf{FP}_{[e,s]} \to \mathsf{FP}_{[e,s]}$ |
| | $+$, $\cdot$, $\div$ | fp.add, fp.mul, fp.div | $\mathsf{RM} \times \mathsf{FP}_{[e,s]} \times \mathsf{FP}_{[e,s]} \to \mathsf{FP}_{[e,s]}$ |
| | fma | fp.fma | $\mathsf{RM} \times \mathsf{FP}_{[e,s]} \times \mathsf{FP}_{[e,s]} \times \mathsf{FP}_{[e,s]} \to \mathsf{FP}_{[e,s]}$ |
| Ints | $+$, $-$ | $+$, $-$ | $\mathsf{Int} \times \mathsf{Int} \to \mathsf{Int}$ |
| Reals | $+$, $-$, $\div$ | $+$, $-$, $/$ | $\mathsf{Real} \times \mathsf{Real} \to \mathsf{Real}$ |

**Table 1.** Set of operators considered in SyGuS grammars.

The (non-constant) operators and their SMT-LIB names and types are listed in Table 1. Note that we further restrict the division operator $\div$ of sort $\mathsf{Real}$ to division by value, i.e., we do not allow division by an arbitrary term of sort $\mathsf{Real}$. We also add a set of special values of the corresponding sort to each default grammar. We represent *bit-vector* values of sort $\mathsf{BV}_{[n]}$ as bit-strings of length $n$, where the left-most bit is the most significant bit. For *floating-point* values of sort $\mathsf{FP}_{[e,s]}$, we use bit strings where the left-most bit indicates the sign, the following $e$ bits represent the exponent, and the remaining bits the significand. For the theory of fixed-size bit-vectors, we use $\text{smax}_{[n]}$ or $\text{smin}_{[n]}$ for the *maximum* or *minimum signed value* of width $n$, e.g., $\text{smax}_{[4]} = 0111$ and $\text{smin}_{[4]} = 1000$, and $\text{ones}_{[n]}$ for the maximum unsigned value, e.g., $\text{ones}_{[4]} = 1111$. For the theory of floating-point numbers, we use $\pm 0$ for *positive* and *negative zero*, $\pm\infty$ for *positive* and *negative infinity*, and NaN for *not a number*, e.g., $-0_{[3,5]} = 10000000$ and $+\infty_{[3,5]} = 01110000$. We further use $\pm\text{min}^s$ for the *positive* and *negative smallest subnormal*, $\pm\text{max}^s$ for the *positive* and *negative largest subnormal*, $\pm\text{min}^n$ for the *positive* and *negative smallest normal*, and $\pm\text{max}^n$ for the *positive* and *negative largest normal*, e.g., $-\text{max}^s_{[3,5]} = 10001111$ and $+\text{min}^n_{[3,5]} = 00010000$. In the definition of grammar $R_{\text{FP}}$ above, we use symbol $\pm$ to indicate that both the positive and negative variant of a special value is included in the grammar.

We extend the above set of default grammars ($\mathbf{grammar}_{\mathcal{S}}$ in Algorithm 1) with ground terms that occur in an input set $\{Q_1, \ldots, Q_n\} \cup G_0$ based on the sort of variable $x \in \boldsymbol{x}$ in $Q_i = \forall \boldsymbol{x}.\, P[\boldsymbol{x}]$ and a *term selection strategy.* This strategy is based on the following two factors. We consider three modes for the *scope* of ground terms: (1) ground terms that occur in quantified formula $Q_i$ (strategy $\mathsf{in}$) (2) ground terms that occur in the set of ground formulas $G$ (strategy $\mathsf{out}$), and (3) the union of (1) and (2) (strategy $\mathsf{both}$). We consider three modes for the *size* of ground terms, defined as the number of subterms a term consists of: (a) terms of *minimal* size, i.e., constants that occur in a term (strategy $\mathsf{min}$) (b) terms of *maximal* size (strategy $\mathsf{max}$), and (c) the union of (a) and (b) (strategy both). For example, for a ground term $a + b \cdot c$, strategy min will select $a, b, c$, max will select $a + b \cdot c$, and both will select $a, b, c, a + b \cdot c$. Each of the scope and size modes may be combined, giving $3 * 3 = 9$ possible term selection strategies.

*Example 1.* Let $Q = \forall x. x \cdot x \not\approx a \cdot a + b \cdot b + 2 \cdot a \cdot b$ where $x, a, b$ have integer type and suppose we run $\mathbf{syqi}(\{Q\}, \emptyset)$. The algorithm first constructs the grammar $\mathbf{grammar}_{\mathcal{S}}(x)$ for $x$, where we assume term selection strategy $\mathcal{S}$ with scope in and size min, which considers ground terms that occur in $Q$ and are of minimal size (2, $a$, and $b$). This grammar is encoded as the following datatype $\mathcal{Z}$:

$$\mathcal{Z} = \mathsf{zero} \mid \mathsf{one} \mid \mathsf{plus}(\mathcal{Z}, \mathcal{Z}) \mid \mathsf{minus}(\mathcal{Z}, \mathcal{Z}) \mid \mathsf{two} \mid \mathsf{a} \mid \mathsf{b}$$

The algorithm introduces a fresh datatype variable $d_x$ of type $\mathcal{Z}$, a fresh integer variable $\mathsf{e}_{d_x}$ of integer type, and adds $l \Rightarrow \mathsf{e}_{d_x} \cdot \mathsf{e}_{d_x} \approx a \cdot a + b \cdot b + 2 \cdot a \cdot b$ to the internal set $G$ of ground formulas, where $l$ is a fresh Boolean variable. In the first iteration of the loop, we have that $G$ (and $G \cup \{l\}$) are satisfiable. Hence, the algorithm calls $\mathbf{select\_lemmas}_{\mathcal{L}}$ on $Q$ and a model $\mathcal{I}$ for $G$; assume that $d_x^{\mathcal{I}} = \mathsf{zero}$ and $\mathsf{e}_{d_x}^{\mathcal{I}} = a^{\mathcal{I}} = b^{\mathcal{I}} = 0$. Based on the lemma selection strategy, we may choose to add the instantiation lemma $0 \cdot 0 \not\approx a \cdot a + b \cdot b + 2 \cdot a \cdot b$, or the evaluation lemma $\mathsf{is}_{\mathsf{zero}}(d_x) \Rightarrow \mathsf{e}_{d_x} \approx 0$, or both lemmas to $G$. Assuming both lemmas are added to $G$, the next iteration of the loop will consider a new model $\mathcal{I}'$ where $d_x^{\mathcal{I}'} \neq \mathsf{zero}$ and $\mathsf{e}_{d_x}^{\mathcal{I}'} \neq 0$. The algorithm will continue finding models with new values for $d_x$, until it finds a model $\mathcal{I}''$ where $d_x^{\mathcal{I}''} = \mathsf{plus}(\mathsf{a}, \mathsf{b})$. At this point the instantiation lemma $(a + b) \cdot (a + b) \not\approx a \cdot a + b \cdot b + 2 \cdot a \cdot b$ will be added to $G$, which is equivalent to false, and **syqi** will terminate with *unsat*. □

### 3.2 Implementation Details

We implemented syntax-guided quantifier instantiation in the CVC4 [5] solver, which has support for a wide range of background theories, covering all those in the SMT-LIB standard library [2]. CVC4 is based on the CDCL($T$) (formerly DPLL($T$)) framework [19]. This framework integrates a propositional SAT solver, which attempts to find a Boolean assignment that propositionally satisfies the input formula, with one or more specialize theory solvers, which monitor the assignments made by the SAT solver to theory literal and flag a conflict if the assignments are ever inconsistent in their theory.

Our SyQI technique is implemented as a module of the subsolver of CVC4 that handles quantified formulas. We leverage CVC4's support for smart enumerative SyGuS as described in Reynolds et al. [22]. Specifically, the **check** method in line 7 in Algorithm 1 involves calling the (combination) of quantifier-free theory solvers, which includes an extension of the theory of datatypes described in the following.

*Symmetry Breaking for Smart Enumerative Synthesis.* As described in previous work [22, 24], CVC4 uses advanced techniques for *symmetry breaking* for the datatypes over which context-free grammars are embedded. The quantifier-free datatype theory solver in CVC4 is extended to issue symmetry blocking clauses based on reasoning about such datatypes, so that the models we generate for a datatype variable $d$ are such that **to_term**$(d)$ is unique with respect to rewriting. For example, the terms $a + b$ and $b + a$ are equivalent, and in CVC4, one will be rewritten to the other. Thus, we know that we only have to consider one variant, e.g., $a + b$. Hence, the extended datatypes solver may issue the blocking clause $\neg \mathsf{is}_{\mathsf{plus}}(d) \vee \neg \mathsf{is}_{\mathsf{b}}(\mathsf{sel}_{\mathcal{Z},1}(d)) \vee \neg \mathsf{is}_{\mathsf{a}}(\mathsf{sel}_{\mathcal{Z},2}(d))$, effectively stating that the term associated with $d$ should not be $b + a$. This technique is highly valuable for syntax-guided synthesis, since it reduces the set of terms considered in the search for candidate solutions. In the context of this work, these techniques are of great importance, since they guarantee that our algorithm does not consider multiple instantiations over tuples of pairwise equivalent terms.

*Quantified Formulas within Boolean Structure and Nested Quantification.* As mentioned earlier, while not shown in Algorithm 1, our approach uses standard techniques for handling qeneral quantified formulas, in particular with quantifiers that occur below Boolean connectives. In the context of CDCL($T$), for each quantified formula $Q_i$ of the form $\forall \boldsymbol{x}.\, P[\boldsymbol{x}]$, the propositional model of our Boolean structure may either assign $Q_i$ to true or false, or leave it unassigned. Quantified formulas that are assigned to false are *Skolemized*, i.e., a lemma of the form $\neg Q_i \Rightarrow \neg P[\boldsymbol{k}]$, where $\boldsymbol{k}$ are fresh constants, is returned to the SAT solver. Quantified formulas that are unassigned are ignored. Quantified formulas that are assigned to true are either active or inactive based on the value assigned to their counterexample literals. Those that are active are processed via **select_lemmas**$_{\mathcal{L}}$. In practice, instantiation lemmas are guarded so that $Q_i \Rightarrow P[\boldsymbol{t}]$ is returned to the SAT solver, meaning that the conclusion only holds when $Q_i$ is assigned to true. Furthermore, each $Q_i$ may have nested quantification, that is, the formula $P$ the counterexample lemma $l_i \Rightarrow \neg P[\mathbf{e}_{\boldsymbol{d_x}}]$ may contain quantified subformulas. Those quantified formulas are then processed by our full algorithm in the same way as quantified formulas from the input.

## 4 Experiments

We implemented our approach in the SMT solver CVC4 [5]. We provide here an extensive evaluation of the techniques and strategies described in Section 3. We first evaluate term and lemma selection strategies for grammar construction, and then compare the performance of our best configuration against Z3 [16], the only state-of-the-art SMT solver besides CVC4 that supports all the logics supported by our implementation.

We performed all experiments on a cluster with Intel Xeon CPU E5-2620 CPUs with 2.1GHz and 128GB memory. We used a time limit of 300 seconds, and an 8GB memory limit for each solver/benchmark pair and count memory out as time out. We evaluate here all configurations on all quantified logics in SMT-LIB [2] that do not contain uninterpreted functions (UF). As an exception, we include the logic UFBV, since the benchmarks in this logic rely

| Strategy | Solved | Sat | Unsat | TO | MO | Uniq | Time[s] |
|---|---|---|---|---|---|---|---|
| Term Selection Strategies | | | | | | | |
| both-max | 12865 | 825 | 12040 | 2871 | 10 | 8 | 886137.3 |
| both-both | 12848 | 823 | 12025 | 2887 | 11 | 12 | 892219.8 |
| both-min | 12843 | 819 | 12024 | 2893 | 10 | 10 | 893808.7 |
| in-both | 12688 | 831 | 11857 | 3052 | 6 | 6 | 939886.7 |
| in-min | 12673 | 828 | 11845 | 3065 | 8 | 4 | 944167.2 |
| in-max | 12667 | 832 | 11835 | 3067 | 12 | 7 | 944952.3 |
| out-both | 12660 | 785 | 11875 | 3081 | 5 | 3 | 948301.4 |
| out-min | 12643 | 788 | 11855 | 3098 | 5 | 2 | 954925.1 |
| out-max | 12616 | 774 | 11842 | 3127 | 3 | 6 | 961683.9 |
| Lemma Selection Strategies | | | | | | | |
| interleave | 12848 | 823 | 12025 | 2887 | 11 | 60 | 892272.2 |
| priority-inst | 12838 | 821 | 12017 | 2893 | 15 | 49 | 897454.3 |
| priority-eval | 12721 | 821 | 11900 | 3019 | 6 | 52 | 938443.4 |

**Table 2.** Selection strategies on considered logics (15,746 benchmarks).

almost entirely on BV reasoning only. We generally exclude logics with UF since for such logics counterexample-guided techniques, as in our approach, are not expected to be more effective than heuristic instantiation techniques such as E-matching, which we confirmed in a preliminary evaluation. Overall, we include logics BV (bit-vectors), FP (floating-point arithmetic), LIA (linear integer arithmetic), LRA (linear real arithmetic), NIA (non-linear integer arithmetic), NRA (non-linear real arithmetic), and their combinations BVFP, BVFPLRA, FPLRA, and UFBV. In total, our benchmark set consists of 15,746 benchmarks.

**Term Selection for Grammar Construction.** As a first experiment, we determine the best combination of scope-based and size-based ground term selection strategies for grammar construction as introduced in Section 3.1. We combine strategies based on scope with strategies based on term size into *nine* selection strategies: in-min, in-max, in-both, out-min, out-max, out-both, both-min, both-max, both-both. The results for our SyGuS instantiation approach with these strategies enabled is shown in Table 2. Note that preliminary experiments identified lemma selection strategy interleave as the best. Hence, we use strategy interleave as the lemma selection strategy for this experiment.

Overall, using strategy both for the scope performs best. Furthermore, for this strategy all three size-based strategies perform equally well. For the remaining experiments, we use strategy both-both as the term selection strategy for grammar construction, where both minimal and maximal ground terms are selected from both the quantified formula $Q_i$ (containing the variable we construct a grammar for) and the set of ground formulas $G$. Note that we choose the more general strategy both-both over strategy both-max even though both-max performs slightly better.

**Lemma Selection.** In our second experiment, we determine the best lemma selection strategy out of the three strategies priority-inst, priority-eval and interleave described in Section 3. The results are shown in Table 2. Note that we use the previously determined best term selection strategy both-both in this experiment.

The best overall strategy is interleave, indicating that it is beneficial to consider instantiation lemmas and evaluation unfolding lemmas in parallel. On the other hand, prioritizing evaluation lemmas over instantiation lemmas (priority-eval) performed significantly worse than the other two configurations. Since this strategy prioritizes evaluation lemmas, it has the advantage over other configurations of delaying instantiations until we obtain an interpretation $\mathcal{I}$ where the interpretation of $\mathsf{e}_{d_x}$ is consistent with respect to $d_x$, i.e., $\mathsf{e}_{d_x}^{\mathcal{I}} = \textbf{to\_term}(d_x)^{\mathcal{I}}$. As a consequence, prioritizing evaluation lemmas puts more effort into finding terms in instantiation that are guaranteed to refine the current candidate model $\mathcal{I}$. However, we conclude from these results that it is often effective to consider instantiations in an eager fashion, either in parallel or even before considering evaluation lemmas. This is likely because instantiation lemmas may often refine the set of possible models even when $G$ does not yet force our evaluation variables to have an interpretation that is consistent with their corresponding datatype values. Nevertheless, we found that evaluation lemmas are often necessary in practice for ensuring our procedure does not get stuck on a single model. When only instantiation lemmas are used, our procedure often terminates the loop with no new lemmas. This is to be expected, as such a strategy violates the requirements for the progress property of Theorem 1.

In the remaining experiment, we use strategy interleave as the lemma selection strategy since it performs slightly better than priority-inst.

**Comparison Against Other Techniques.** Finally, we compare our SyGuS instantiation approach against other techniques implemented in CVC4, the state-of-the-art SMT solvers Z3 [16] (version 4.8.9) and Boolector [17] (version 3.2.1), and the superposition-based theorem prover Vampire [13] (version 4.5.1). Note that Boolector implements counterexample-guided model synthesis [20] but only supports the SMT-LIB logic BV, whereas Vampire supports LIA, LRA, NIA, and NRA. We consider the following four configurations of CVC4: **ematch**: with E-matching [15] enabled; **cegqi**: with CEGQI for linear arithmetic [25] and bit-vectors [18] enabled, falls back to value-based instantiation techniques for other theories; **enum**: with enumerative instantiation [21] enabled; **syqi**: with our SyGuS instantiation approach enabled. We use strategy both-both for term selection, and interleave for lemma selection.

The results are summarized in Table 3. First, note that Z3 disagrees on 10 benchmarks in logic FP with the other four CVC4 configurations. This is due to a known problem in Z3 related to operator rem, where it answers *sat* instead of *unsat*. We do not count these 10 benchmarks as solved and give the number of disagreements in parenthesis marked with a * in Table 3.

Overall, note that E-matching (**ematch**) performs very poorly on these benchmark sets. This is not surprising since it is designed with a focus on problems with uninterpreted functions. To a lesser extent, enumerative instantiation (**enum**) also performs poorly, probably also due to the fact that it is not designed for inputs without uninterpreted functions. In detail, both this configuration and

| Logic | | syqi | cegqi | ematch | enum | Z3 | Boolector | Vampire |
|---|---|---|---|---|---|---|---|---|
| BV | sat | 269 | 411 | 203 | 204 | 566 | 620 | - |
| (5846) | unsat | 4752 | 5039 | 3846 | 4699 | 4934 | 4889 | - |
| | unsolved | 825 | 396 | 1797 | 943 | 346 | 337 | - |
| BVFP | sat | 113 | 110 | 26 | 29 | 174 | - | - |
| (224) | unsat | 14 | 4 | 4 | 14 | 11 | - | - |
| | unsolved | 97 | 110 | 194 | 181 | 39 | - | - |
| BVFPLRA | sat | 103 | 95 | 67 | 67 | 164 | - | - |
| (185) | unsat | 5 | 5 | 5 | 6 | 5 | - | - |
| | unsolved | 77 | 85 | 113 | 112 | 16 | - | - |
| FP | sat | 34 | 28 | 23 | 23 | 47 | - | - |
| (2484) | unsat | 2117 | 1899 | 83 | 1615 | 1923 | - | - |
| | unsolved | 333 | 557 | 2378 | 846 | 504 (10)* | - | - |
| FPLRA | sat | 17 | 17 | 13 | 13 | 18 | - | - |
| (27) | unsat | 0 | 0 | 0 | 0 | 0 | - | - |
| | unsolved | 10 | 10 | 14 | 14 | 9 | - | - |
| LIA | sat | 188 | 199 | 19 | 19 | 189 | - | 5 |
| (607) | unsat | 319 | 357 | 46 | 171 | 295 | - | 310 |
| | unsolved | 100 | 51 | 542 | 417 | 123 | - | 292 |
| LRA | sat | 79 | 593 | 461 | 461 | 740 | - | 0 |
| (2419) | unsat | 955 | 1306 | 1018 | 1117 | 1454 | - | 871 |
| | unsolved | 1385 | 520 | 940 | 841 | 225 | - | 1548 |
| NIA | sat | 12 | 11 | 6 | 6 | 12 | - | 0 |
| (20) | unsat | 7 | 8 | 1 | 5 | 5 | - | 6 |
| | unsolved | 1 | 1 | 13 | 9 | 3 | - | 14 |
| NRA | sat | 0 | 0 | 0 | 0 | 2 | - | 0 |
| (3813) | unsat | 3781 | 3781 | 3703 | 3768 | 3806 | - | 3803 |
| | unsolved | 32 | 32 | 110 | 45 | 5 | - | 10 |
| UFBV | sat | 8 | 8 | 8 | 8 | 26 | - | - |
| (121) | unsat | 74 | 53 | 47 | 66 | 72 | - | - |
| | unsolved | 39 | 60 | 66 | 47 | 23 | - | - |
| **Total** | **sat** | **823** | **1472** | **826** | **830** | **1938** | - | - |
| (15746) | **unsat** | **12024** | **12452** | **8753** | **11461** | **12505** | - | - |
| | **unsolved** | **2899** | **1822** | **6167** | **3455** | **1293 (10)*** | - | - |

**Table 3.** SyQI vs. other techniques, Z3, Boolector, and Vampire (15,746 benchmarks).

**syqi** are enumerative in nature. The former uses a selection strategy based on the evolving ground terms in the current context, whereas the latter uses a fixed grammar built from the initial set of terms. In a sense, **syqi** leverages the power of a grammar for discovering new terms, whereas **enum** adapts to what terms are generated by instantiations. Overall, **syqi** solves 556 more benchmarks than enumerative instantiation, justifying the need for a syntax-guided approach for instantiation for inputs that are rich in background theories.

Our results show that **syqi** is remarkably competitive when compared to **cegqi**, which uses the best known theory-specific instantiation strategies. The performance of syntax-guided instantiation matches or exceeds counterexample-guided instantiation on logics BVFP, BVFPLRA, FP, FPLRA, NIA, NRA, and UFBV. In particular, for quantified floating-point arithmetic (FP), the performance of **syqi** significantly outperforms **cegqi**, where it solves 224 more benchmarks. We attribute this to the fact that **cegqi** only performs value-based instantiation, whereas the use of grammars is effective in determining useful symbolic terms to use in instantiations for this theory. Interestingly, **syqi** solves the only satisfiable benchmark in the NIA category that is unsolved by **cegqi**, meaning that in a portfolio setting with all available configurations, CVC4 solves all benchmarks in this category. On the other hand, counterexample-guided instantiation outperforms **syqi** on logics such as LIA, LRA, and BV, where well-established instantiation strategies exist. Syntax-guided techniques are especially ineffective for linear real arithmetic, since it is often important to construct specific real constants based on solving sets of linear (in)equalities [25].

Comparing all configurations of CVC4 with Z3, Boolector, and Vampire, we see that in some logics like LIA and NIA, counterexample-guided instantiation in CVC4 outperforms Z3 and Vampire, whereas in other logics like NRA, UFBV, and many logics that combine BV, FP and LRA, Z3 performs best. For the logic BV, Boolector outperforms CVC4 and Z3; however, CVC4 solves the most unsatisfiable instances. The **syqi** configuration performs best on the floating-point benchmarks, where it solves 181 more than the closest competitor. When comparing the four CVC4 configurations in terms of uniquely solved instances, **cegqi** uniquely solves 660 instances, **syqi** 119 instances, **enum** 117 instances, and **ematch** not a single one. Between configurations **cegqi** and **syqi**, the former uniquely solves 1479 instances, and the latter 402 instances.

In summary, theory-specific approaches as implemented in CVC4, Z3, and Boolector outperform **syqi** in categories where instantiation strategies are highly mature, such as linear integer and real arithmetic, and fixed-width bit-vectors. Nevertheless, our evaluation demonstrates the versatility of the approach, especially for benchmarks using quantified floating-point arithmetic or combined theories where no good approach to quantifier instantiation was known.

## 5 Conclusion

We have presented a syntax-guided approach for quantifier instantiation and implemented it in the SMT solver CVC4. Our experiments show that our approach is a viable alternative to theory-specific quantifier instantiation techniques and can be applied to a wide range of logics. In particular, for the theory of floating-point arithmetic, syntax-guided instantiation in CVC4 significantly outperforms the state of the art. In future work, we plan to tune our grammar construction based on an analysis of which terms are more likely to appear in conflicts, which can potentially be done automatically. Another direction of future work is to provide an interface that would allow users to supply their own grammars for use in SyQI, similarly to the user-provided triggers for E-matching. We also plan to use our approach as a baseline for quantified logics in recent (and future) new theories. Currently, support in SMT solvers is highly limited, for instance, for quantified formulas involving the theory of strings and regular expressions. Syntax-guided instantiation can serve as a baseline for potential user applications that rely on quantified formulas in these theories.

## References

[1] Alur, R., Bodík, R., Juniwal, G., Martin, M.M.K., Raghothaman, M., Seshia, S.A., Singh, R., Solar-Lezama, A., Torlak, E., Udupa, A.: Syntax-guided synthesis. In: Formal Methods in Computer-Aided Design, FMCAD 2013, Portland, OR, USA, October 20–23, 2013. pp. 1–8. IEEE (2013), http://ieeexplore.ieee.org/document/6679385/

[2] Barrett, C., Fontaine, P., Tinelli, C.: The Satisfiability Modulo Theories Library (SMT-LIB) (2020), http://www.SMT-LIB.org

[3] Barrett, C., Shikanian, I., Tinelli, C.: An abstract decision procedure for satisfiability in the theory of recursive data types. Electr. Notes Theor. Comput. Sci. **174**(8), 23–37 (2007). 10.1016/j.entcs.2006.11.037

[4] Barrett, C., Stump, A., Tinelli, C.: The SMT-LIB Standard: Version 2.0. In: Gupta, A., Kroening, D. (eds.) Proceedings of the 8th International Workshop on Satisfiability Modulo Theories (Edinburgh, UK) (2010)

[5] Barrett, C.W., Conway, C.L., Deters, M., Hadarean, L., Jovanovic, D., King, T., Reynolds, A., Tinelli, C.: CVC4. In: Gopalakrishnan, G., Qadeer, S. (eds.) Computer Aided Verification - 23rd International Conference, CAV 2011, Snowbird, UT, USA, July 14–20, 2011. Proceedings. Lecture Notes in Computer Science, vol. 6806, pp. 171–177. Springer (2011). 10.1007/978-3-642-22110-1_14

[6] Bjørner, N., Janota, M.: Playing with quantified satisfaction. In: Fehnker, A., McIver, A., Sutcliffe, G., Voronkov, A. (eds.) 20th International Conferences on Logic for Programming, Artificial Intelligence and Reasoning - Short Presentations, LPAR 2015, Suva, Fiji, November 24–28, 2015. EPiC Series in Computing, vol. 35, pp. 15–27. EasyChair (2015), https://easychair.org/publications/paper/jmM

[7] Brain, M., Niemetz, A., Preiner, M., Reynolds, A., Barrett, C.W., Tinelli, C.: Invertibility conditions for floating-point formulas. In: Dillig, I., Tasiran, S. (eds.) Computer Aided Verification - 31st International Conference, CAV 2019, New York City, NY, USA, July 15–18, 2019, Proceedings, Part II. Lecture Notes in Computer Science, vol. 11562, pp. 116–136. Springer (2019). 10.1007/978-3-030-25543-5_8

[8] Detlefs, D., Nelson, G., Saxe, J.B.: Simplify: a theorem prover for program checking. J. ACM **52**(3), 365–473 (2005). 10.1145/1066100.1066102

[9] Ferrante, J., Rackoff, C.: A decision procedure for the first order theory of real addition with order. SIAM J. Comput. **4**(1), 69–76 (1975). 10.1137/0204006

[10] Ge, Y., Barrett, C.W., Tinelli, C.: Solving quantified verification conditions using satisfiability modulo theories. In: Pfenning, F. (ed.) Automated Deduction - CADE-21, 21st International Conference on Automated Deduction, Bremen, Germany, July 17–20, 2007, Proceedings. Lecture Notes in Computer Science, vol. 4603, pp. 167–182. Springer (2007). 10.1007/978-3-540-73595-3_12

[11] Ge, Y., de Moura, L.M.: Complete instantiation for quantified formulas in satisfiabiliby modulo theories. In: Bouajjani, A., Maler, O. (eds.) Computer Aided Verification, 21st International Conference, CAV 2009, Grenoble, France, June 26 - July 2, 2009. Proceedings. Lecture Notes in Computer Science, vol. 5643, pp. 306–320. Springer (2009). 10.1007/978-3-642-02658-4_25

[12] K., H.G.V., Fedyukovich, G., Gurfinkel, A.: Word level property directed reachability. In: IEEE/ACM International Conference On Computer Aided Design, ICCAD 2020, San Diego, CA, USA, November 2–5, 2020. pp. 107:1–107:9. IEEE (2020). 10.1145/3400302.3415708

[13] Kovács, L., Voronkov, A.: First-order theorem proving and vampire. In: Sharygina, N., Veith, H. (eds.) Computer Aided Verification - 25th International Conference, CAV 2013, Saint Petersburg, Russia, July 13–19, 2013. Proceedings. Lecture Notes in Computer Science, vol. 8044, pp. 1–35. Springer (2013). 10.1007/978-3-642-39799-8_1

[14] Loos, R., Weispfenning, V.: Applying linear quantifier elimination. Comput. J. **36**(5), 450–462 (1993). 10.1093/comjnl/36.5.450

[15] de Moura, L.M., Bjørner, N.: Efficient e-matching for SMT solvers. In: Pfenning, F. (ed.) Automated Deduction - CADE-21, 21st International Conference on Automated Deduction, Bremen, Germany, July 17–20, 2007, Proceedings. Lecture Notes in Computer Science, vol. 4603, pp. 183–198. Springer (2007). 10.1007/978-3-540-73595-3_13

[16] de Moura, L.M., Bjørner, N.: Z3: an efficient SMT solver. In: Ramakrishnan, C.R., Rehof, J. (eds.) Tools and Algorithms for the Construction and Analysis of Systems, 14th International Conference, TACAS 2008, Held as Part of the Joint European Conferences on Theory and Practice of Software, ETAPS 2008, Budapest, Hungary, March 29-April 6, 2008. Proceedings. Lecture Notes in Computer Science, vol. 4963, pp. 337–340. Springer (2008). 10.1007/978-3-540-78800-3_24

[17] Niemetz, A., Preiner, M., Biere, A.: Boolector 2.0. J. Satisf. Boolean Model. Comput. **9**(1), 53–58 (2014). 10.3233/sat190101

[18] Niemetz, A., Preiner, M., Reynolds, A., Barrett, C.W., Tinelli, C.: On solving quantified bit-vector constraints using invertibility conditions. Formal Methods in System Design pp. 1572–8102 (2021). 10.1007/s10703-020-00359-9

[19] Nieuwenhuis, R., Oliveras, A., Tinelli, C.: Solving SAT and SAT Modulo Theories: from an abstract Davis-Putnam-Logemann-Loveland Procedure to DPLL(T). Journal of the ACM **53**(6), 937–977 (2006)

[20] Preiner, M., Niemetz, A., Biere, A.: Counterexample-guided model synthesis. In: Legay, A., Margaria, T. (eds.) Tools and Algorithms for the Construction and Analysis of Systems - 23rd International Conference, TACAS 2017, Held as Part of the European Joint Conferences on Theory and Practice of Software, ETAPS 2017, Uppsala, Sweden, April 22–29, 2017, Proceedings, Part I. Lecture Notes in Computer Science, vol. 10205, pp. 264–280 (2017). 10.1007/978-3-662-54577-5_15

[21] Reynolds, A., Barbosa, H., Fontaine, P.: Revisiting enumerative instantiation. In: Beyer, D., Huisman, M. (eds.) Tools and Algorithms for the Construction and Analysis of Systems - 24th International Conference, TACAS 2018, Held as Part of the European Joint Conferences on Theory and Practice of Software, ETAPS 2018, Thessaloniki, Greece, April 14–20, 2018, Proceedings, Part II. Lecture Notes in Computer Science, vol. 10806, pp. 112–131. Springer (2018). 10.1007/978-3-319-89963-3_7

[22] Reynolds, A., Barbosa, H., Nötzli, A., Barrett, C.W., Tinelli, C.: cvc4sy: Smart and fast term enumeration for syntax-guided synthesis. In: Dillig, I., Tasiran, S. (eds.) Computer Aided Verification - 31st International Conference, CAV 2019, New York City, NY, USA, July 15–18, 2019, Proceedings, Part II. Lecture Notes in Computer Science, vol. 11562, pp. 74–83. Springer (2019). 10.1007/978-3-030-25543-5_5

[23] Reynolds, A., Blanchette, J.C.: A decision procedure for (co)datatypes in SMT solvers. In: Felty, A.P., Middeldorp, A. (eds.) Automated Deduction - CADE-25 - 25th International Conference on Automated Deduction, Berlin, Germany, August 1–7, 2015, Proceedings. Lecture Notes in Computer Science, vol. 9195, pp. 197–213. Springer (2015). 10.1007/978-3-319-21401-6_13, 10.1007/978-3-319-21401-6_13

[24] Reynolds, A., Deters, M., Kuncak, V., Tinelli, C., Barrett, C.W.: Counterexample-guided quantifier instantiation for synthesis in SMT. In: Kroening, D., Pasareanu, C.S. (eds.) Computer Aided Verification - 27th International Conference, CAV 2015, San Francisco, CA, USA, July 18–24, 2015, Proceedings, Part II. Lecture Notes in Computer Science, vol. 9207, pp. 198–216. Springer (2015). 10.1007/978-3-319-21668-3_12

[25] Reynolds, A., King, T., Kuncak, V.: Solving quantified linear arithmetic by counterexample-guided instantiation. Formal Methods Syst. Des. **51**(3), 500–532 (2017). 10.1007/s10703-017-0290-y

[26] Reynolds, A., Tinelli, C., Goel, A., Krstic, S., Deters, M., Barrett, C.W.: Quantifier instantiation techniques for finite model finding in SMT. In: Bonacina, M.P. (ed.) Automated Deduction - CADE-24 - 24th International Conference on Automated Deduction, Lake Placid, NY, USA, June 9–14, 2013. Proceedings. Lecture Notes in Computer Science, vol. 7898, pp. 377–391. Springer (2013). 10.1007/978-3-642-38574-2_26

[27] Reynolds, A., Tinelli, C., de Moura, L.M.: Finding conflicting instances of quantified formulas in SMT. In: Formal Methods in Computer-Aided Design, FMCAD 2014, Lausanne, Switzerland, October 21–24, 2014. pp. 195–202. IEEE (2014). 10.1109/FMCAD.2014.6987613

[28] Reynolds, A., Viswanathan, A., Barbosa, H., Tinelli, C., Barrett, C.: Datatypes with shared selectors. In: Automated Reasoning - 9th International Joint Conference, IJCAR 2018, Held as Part of the Federated Logic Conference, FloC 2018, Oxford, UK, July 14–17, 2018, Proceedings. pp. 591–608 (2018). 10.1007/978-3-319-94205-6_39

[29] Wintersteiger, C.M., Hamadi, Y., de Moura, L.M.: Efficiently solving quantified bit-vector formulas. In: Bloem, R., Sharygina, N. (eds.) Proceedings of 10th International Conference on Formal Methods in Computer-Aided Design, FMCAD 2010, Lugano, Switzerland, October 20–23. pp. 239–246. IEEE (2010), http://ieeexplore.ieee.org/document/5770955/

